# Molecular Mechanisms of Neurodegeneration Related to *C9orf72* Hexanucleotide Repeat Expansion

**DOI:** 10.1155/2019/2909168

**Published:** 2019-01-15

**Authors:** Mirjana Babić Leko, Vera Župunski, Jason Kirincich, Dinko Smilović, Tibor Hortobágyi, Patrick R. Hof, Goran Šimić

**Affiliations:** ^1^Department of Neuroscience, Croatian Institute for Brain Research, University of Zagreb School of Medicine, Zagreb, Croatia; ^2^Department of Biochemistry, Faculty of Chemistry and Chemical Technology, University of Ljubljana, Ljubljana, Slovenia; ^3^Institute of Pathology, Faculty of Medicine, University of Szeged, Szeged, Hungary; ^4^MTA-DE Cerebrovascular and Neurodegenerative Research Group, University of Debrecen, Debrecen, Hungary; ^5^Fishberg Department of Neuroscience, Friedman Brain Institute, Ronald M. Loeb Center for Alzheimer's Disease, Icahn School of Medicine at Mount Sinai, New York, USA

## Abstract

Two clinically distinct diseases, amyotrophic lateral sclerosis (ALS) and frontotemporal dementia (FTD), have recently been classified as two extremes of the FTD/ALS spectrum. The neuropathological correlate of FTD is frontotemporal lobar degeneration (FTLD), characterized by tau-, TDP-43-, and FUS-immunoreactive neuronal inclusions. An earlier discovery that a hexanucleotide repeat expansion mutation in chromosome 9 open reading frame 72 (*C9orf72*) gene causes ALS and FTD established a special subtype of ALS and FTLD with TDP-43 pathology (C9FTD/ALS). Normal individuals carry 2–10 hexanucleotide GGGGCC repeats in the *C9orf72* gene, while more than a few hundred repeats represent a risk for ALS and FTD. The proposed molecular mechanisms by which *C9orf72* repeat expansions induce neurodegenerative changes are C9orf72 loss-of-function through haploinsufficiency, RNA toxic gain-of-function, and gain-of-function through the accumulation of toxic dipeptide repeat proteins. However, many more cellular processes are affected by pathological processes in C9FTD/ALS, including nucleocytoplasmic transport, RNA processing, normal function of nucleolus, formation of membraneless organelles, translation, ubiquitin proteasome system, Notch signalling pathway, granule transport, and normal function of TAR DNA-binding protein 43 (TDP-43). Although the exact molecular mechanisms through which *C9orf72* repeat expansions account for neurodegeneration have not been elucidated, some potential therapeutics, such as antisense oligonucleotides targeting hexanucleotide GGGGCC repeats in mRNA, were successful in preclinical trials and are awaiting phase 1 clinical trials. In this review, we critically discuss each proposed mechanism and provide insight into the most recent studies aiming to elucidate the molecular underpinnings of C9FTD/ALS.

## 1. Introduction

**Figure 1:**
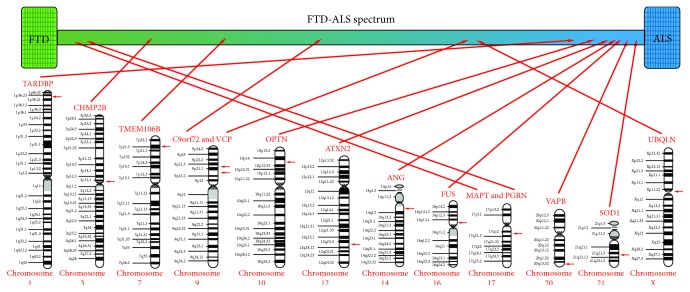
Influence of different genes on FTD/ALS clinical spectrum.

Amyotrophic lateral sclerosis (ALS) and frontotemporal dementia (FTD) are two clinically distinct entities. ALS, also known as motor neuron disease (MND), Lou Gehrig's disease, and Charcot disease, affects both upper and lower motor neurons leading to hyperreflexia, spasticity, fasciculations, and muscular atrophy [1]. The disease onset is mostly after 60 years of age with a prevalence of around 5 cases per 100,000 [2]. ALS patients can be divided into subgroups according to neuropsychological deficits: ALS with cognitive impairment (ALS-ci), ALS with behavioral impairment (ALS-bi), and ALS with combined cognitive and behavioral impairment (ALS-cbi) [3]. In FTD, neurodegeneration affects the frontal and temporal lobes causing frontotemporal lobar degeneration (FTLD) and is associated with changes in behavior and personality, deficits in frontal executive functions, and language impairment [3]. FTD is considered to be one of the most common forms of dementia in the population under 65 years of age with the prevalence around 10 cases per 100,000 [4]. FTD patients are divided in three clinical syndromes according to their symptomatology: two language variants (progressive nonfluent aphasia (PNFA) and semantic dementia (SD)), and behavioral variant of frontotemporal dementia (bvFTD) [3]. Based on the clinical, genetic, and neuropathological overlap between ALS and FTD, these two diseases are now considered as two extremes of the FTD/ALS spectrum (Figure 1) [1]. About 15% of FTD patients show symptoms of ALS, and up to 50% of ALS patients have symptoms of FTD [5].

## 2. Main Features of ALS and FTLD-TDP Neuropathology

The vast majority of ALS cases (ALS-TDP) and the most common FTD pathological subtype (FTLD-TDP) show TDP-43 (TAR DNA-binding protein 43) immunoreactive aggregates forming characteristic inclusions (for overview see [6, 7]). The hallmarks of ALS with TDP-43 pathology (ALS-TDP) are skein-like, granular, and compact inclusions in motor neurons, whereas in FTLD-TDP, there are four distinct morphological types (A–D) with characteristic distribution and morphology of dystrophic neurites, cytoplasmic, and intranuclear inclusions. There is a good correlation with both clinical phenotype and genetic alterations [8]. For example, cases with hexanucleotide repeat expansions in *C9orf72* are typically associated with type A and type B pathology, whereas mutations in the progranulin gene *GRN* and valosin-containing protein gene (*VCP*) are associated with type A and type D, respectively. Regarding the correlation with clinical phenotypes, for example, PNFA is associated with type A, SD with type C, and inclusion body myopathy with frontotemporal dementia (IBMFD) with type D. In both ALS and FTLD, there is glial pathology mainly affecting oligodendroglia, with TDP-43-immunoreactive cytoplasmic inclusions [6, 7]. While most of the ALS cases are sporadic, about half of all FTD patients show a familial pattern of inheritance linked to mutations in several different genes [6, 9–11].

## 3. Hexanucleotide Repeat Expansion in *C9orf72* Gene

A mutation in chromosome 9 open reading frame 72 (*C9orf72*) has been identified as a common genetic cause of both ALS and FTD. The link of ALS and FTD to chromosome 9 was first reported in 2006 in two independent studies [12, 13]. It was later shown that the mutation is an expansion of GGGGCC (G4C2) hexanucleotide repeat in a noncoding region of the *C9orf72* gene [14–16], which is the most common genetic cause of ALS and FTD (so called C9FTD/ALS) in certain populations.

In humans, three transcript variants of *C9orf72* have been identified. Transcript variants 2 and 3 give rise to identical protein isoforms of 481 amino acids, while the 3′ site of transcript variant 1 is truncated and encodes an isoform of 222 amino acids. The hexanucleotide repeat is located within the promoter region for transcript variant 2, which corresponds to the position of the first intron in transcript variants 1 and 3 depending on the transcription start site used [17, 18].

While the number of G4C2 repeat units in the DNA of healthy individuals is up to 25, the number of repeats in the DNA of ALS and FTD patients is usually 400 to several thousand [14–16, 19–21]. A small percentage of patients have shorter expansions, from 45–80 repeats [19], and an even shorter expansion, around 30 repeats, has been associated with the disease [15]. Notably, there is an apparent gap between short pathogenic repeat sizes of 45 to 80 and long expansions from 400 to several thousand units. This is likely due to high genomic instability of the intermediate long repeats, which may have a tendency to either expand or contract [19]. Interestingly, longer expansions have been recently correlated with an earlier onset of disease [19]. In contrast, other studies detected either positive correlation [22–25] or no association [22, 25] between disease severity and expansion size. Additionally, the results of these studies were variable depending on the tissue in which expansion size was measured [22, 25].

## 4. C9orf72 Protein

The function of C9orf72 protein is still unclear. Bioinformatics predictions suggest that C9orf72 belongs to the family of DENN (differentially expressed in normal and neoplastic cells) proteins, which function as RabGEF (guanine exchange factor) regulators of membrane trafficking by activating RabGTPases [26, 27]. RabGEF facilitates a release of GDP from Rab and exchanges it for GTP. To support the role of C9orf72 as RabGEF, a study conducted on neuronal cell lines and human spinal cord tissue revealed that it was colocalized and coprecipitated with Rab proteins [28]. Therefore, C9orf72 could be involved in Rab-mediated cellular trafficking and protein degradation. Additionally, C9orf72 protein was detected at presynaptic sites, and as it interacts with Rab proteins, it is believed that C9orf72 could regulate synaptic vesicles as RabGEF for RAB3 proteins [29]. Xiao et al. detected interactions of C9orf72 with different components of the nuclear pore complex (NPC) and nuclear receptors in human brain tissue, indicating a possible role of C9orf72 in nucleocytoplasmic trafficking [30]. Nuclear transport is compromised in different C9FTD/ALS models (such as *Drosophila*, induced pluripotent stem cells (iPSC) derived neurons, human brain tissue, yeast cells, mouse primary neurons, and primary human dermal fibroblasts) [31–33]. As knockdown of C9orf72 leads to disruption in endosomal trafficking and formation of autophagosome [28], it is proposed that C9orf72 may be also involved in the regulation of endosomal trafficking and autophagy. Additional studies established the involvement of C9orf72 in the regulation of autophagy [34–36]. In studies conducted on cell lines, it was also shown that C9orf72 could be involved in the regulation of lysosomal function [37, 38] and in the formation of stress granules [39]. In mice, C9orf72 is required for the normal macrophage and microglial function [40]. It was shown that decreased levels of C9orf72 cause dysfunctional microglia that are related to neurodegeneration [40].

## 5. Neuropathological Features of C9FTD/ALS

Cases with hexanucleotide repeat expansion in *C9orf72* are typically associated with type A and B FTLD-TDP pathology, as previously mentioned. The neuropathology of C9FTD/ALS shows pathognomonic ubiquitin- and p62-positive and, rarely, TDP-43-containing inclusions in the cerebellum (Purkinje cells and granular cells) and hippocampus [41, 42]. A unique feature is the presence of aggregating dipeptide repeat proteins within a proportion of inclusion bodies, some of which may not show phosphorylated TDP-43 (pTDP-43) immunoreactivity [43, 44]. Ultrastructurally, the characteristic inclusions in FTLD and ALS with C9orf72 mutation are granular and filamentous [45].

## 6. Potential Mechanisms of *C9orf72* Hexanucleotide Repeat Expansion-Mediated Neurodegeneration

**Figure 2:**
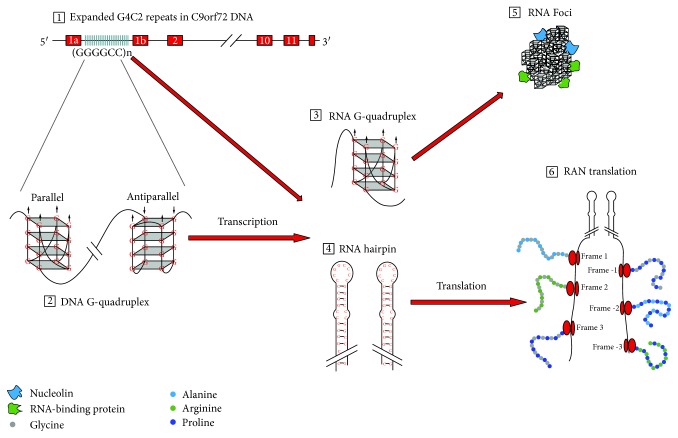
Potential mechanisms of *C9orf72* hexanucleotide repeat expansion (HRE)-mediated neurodegeneration. Pathology due to repeats in *C9orf72* gene may emerge from C9orf72 haploinsufficiency, RNA toxicity, and DPR accumulation. HRE in the noncoding region of the *C9orf72* gene (1) form G-quadruplex structures (2). RNA transcribed from HRE DNA region can form different structures including G-quadruplexes (3) and RNA hairpins (4). HRE-containing RNA form RNA foci (5), which bind RNA-binding proteins. The last possible mechanism underlying pathology in C9FTD/ALS is through the repeat-associated non-ATG (RAN) translation, in which five different dipeptide repeat proteins can be formed—poly-GA, poly-GP, and poly-GR from the sense strand and poly-GP, poly-PA, and poly-PR from the antisense strand (6).

**Figure 3:**
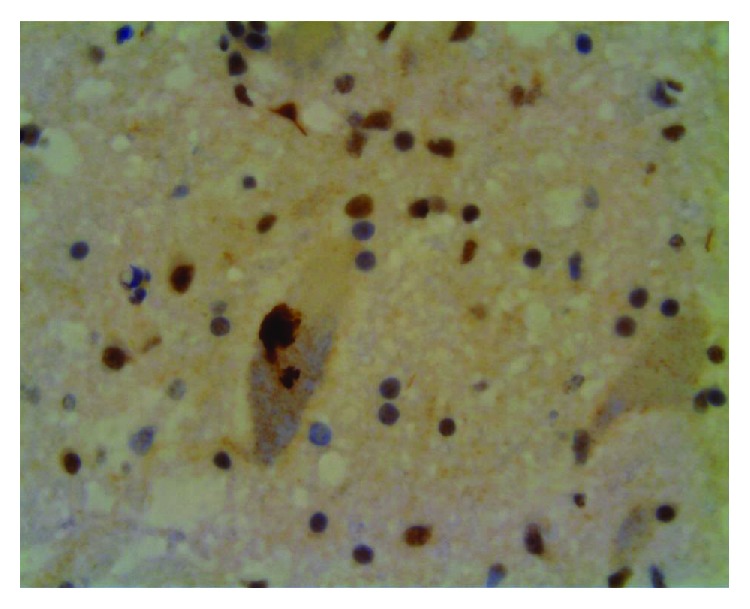
TDP-43-immunoreactive cytoplasmic inclusions, finely granular aggregates, and lack of nuclear labelling in a spinal cord motoneuron of a patient with ALS caused by *C9orf72* hexanucleotide repeat expansion.

Three mechanisms have been proposed for G4C2 hexanucleotide repeat expansion (HRE) in *C9orf72* to induce neurodegenerative changes: (1) loss of C9orf72 function through haploinsufficiency, (2) toxic gain-of-function due to the generation of aberrant HRE-containing RNA, and (3) toxic gain-of-function through the accumulation of dipeptide repeat proteins (DPR) translated from hexanucleotide repeat RNA. Potential mechanisms by which *C9orf72* repeat expansions result in neurodegeneration are summarized in Figure 2. Microphotograph with characteristic histopathological changes is given in Figure 3. Studies investigating these mechanisms are summarized in Table 1.

### 6.1. Loss of C9orf72 Function through Haploinsufficiency

Carriers of *C9orf72* HRE have decreased levels of *C9orf72* transcripts [14, 16], which are also reflected in the decreased C9orf72 protein levels in the frontal and temporal cortex [20], presumably due to the loss of transcription from the mutant allele carrying the HRE. A systematic study of *C9orf72* levels in patients with HRE revealed decreased levels of *C9orf72* transcripts in comparison to non-HRE patients and controls [46]. Interestingly, the levels of the long C9orf72 protein isoform were decreased in the brain, while levels of the short C9orf72 protein isoform were increased (as detected by Western blot). Immunohistochemical analysis of spinal motor neurons showed decreased expression of short C9orf72 protein isoform on nuclear membrane in C9ALS cases compared to controls, while subcellular localization of long C9orf72 protein isoform was unchanged [30]. Another study detected 80% reduction of long C9orf72 protein isoform in the cerebellum of *C9orf72* HRE carriers in comparison to controls [29]. *C9orf72* hexanucleotide repeat expansion might induce DNA hypermethylation and consequently lead to decreased *C9orf72* transcription [47]. HRE is methylated when the number of repeats is larger than 90 [48]. Moreover, the DNA from a fraction of *C9orf72* HRE carriers is also methylated in the 5′ CpG island [47, 49, 50]. The repeat expansion-induced DNA methylation in the *C9orf72* promoter region, which results in downregulated *C9orf72* transcription, therefore provides a likely explanation for the association between the size of repeat expansion and the age of onset of the disease [19]. More precisely, the authors tested *C9orf72* promoter activity in human kidney and neuroblastoma cell lines and observed reduced *C9orf72* transcription in cells with larger repeats and increased methylation. Thus, they proposed that higher methylation of *C9orf72* promoter may be an explanation of how repeat expansion could lead to loss-of-function, without excluding the possibility that repeat expansion could also lead to toxic gain-of-function [19]. There is also a possibility that higher-order DNA structures formed on G4C2 repeats could lead to abortive transcription of *C9orf72* and therefore be the cause for decreased *C9orf72* transcription [51].

C9orf72 loss-of-function in *C. elegans* and zebrafish models results in motor neuron degeneration, indicating a possible disease mechanism [52, 53]. However, human *C9orf72* gene has only partial homology with its ortholog in *C. elegans* and zebrafish. Thus, loss-of-function mechanisms were assessed in *C9orf72* knockout mice as the mouse ortholog of *C9orf72* gene is more similar to the human *C9orf72* gene [18]. Various knockout and knockdown models have been developed, yet none of the mouse models showed phenotypes characteristic for ALS or FTD [35, 40, 54–57], indicating that C9orf72 loss-of-function is not the true cause of disease. The observation that patients homozygous for *C9orf72* repeat expansion do not have more severe symptoms of disease compared to heterozygotes further supports these findings [58, 59] (Table 2).

### 6.2. Toxic Gain-of-Function due to Aberrant HRE-Containing RNA

#### 6.2.1. Higher-Order Structures of DNA and RNA Formed by *C9orf72* HRE Sequence

Due to the uniformity of the G4C2 sequence and the abundance of guanine nucleotides, expanded hexanucleotide repeats in the *C9orf72* gene form higher-order DNA structures called G-quadruplexes [51, 60]. G-quadruplexes formed in the DNA can adopt both parallel- and antiparallel-stranded conformations, and increasing the length of the repeats creates a heterogeneous mixture of these structures. Both C-rich sense and antisense strands can assemble as i-motifs and hairpin structures [61]. G-quadruplex structures formed in the *C9orf72* HRE region cause generation of truncated RNA transcripts that are aborted in the hexanucleotide repeat region [51]. These aberrant RNA transcripts containing repetitive hexanucleotide sequence can also form G-quadruplexes and RNA hairpin structures. Additionally, HRE-containing RNA can form hybrids with HRE-containing DNA called R-loops [51, 62–64]. Together, these higher-order structures of DNA and RNA are thought to act as promoters and regulatory elements affecting replication, transcription, and translation of the surrounding region [65, 66], and to exert deleterious effect on cells by causing nucleolar stress and impeding RNA processing [51], as discussed below.

#### 6.2.2. Sequestration of RNA-Binding Proteins into Nuclear Aggregates by the *C9orf72* HRE-RNA

HRE-containing RNA transcripts accumulate and form nuclear aggregates, or RNA foci, in the brain of patients with *C9orf72* HRE [14]. As the transcription of *C9orf72* HRE DNA region can also occur in the antisense direction, antisense RNA is also found within foci in the brains of patients with *C9orf72* HRE [67, 68]. Several proteins specifically bind to *C9orf72* HRE-containing RNA, including ADARB2, ALYREF, hnRNP H, hnRNP A1, nucleolin, Pur *α*, and SRSF2 [51, 69–73]. A number of RNA-binding proteins (RBP) are implicated in the HRE-induced neurodegeneration.

*(1) Nucleolin*. One of the main constituents of the nucleolus, nucleolin, has a high affinity for *C9orf72* RNA containing G-quadruplex structures of G4C2 repeat [51]. Nucleolin bound to RNA G-quadruplexes dislocates from the nucleoli and disperses through the nucleus. This results in impaired rRNA processing, followed by decreased maturation of ribosomes, and finally, accumulation of untranslated mRNA in the neuronal cytoplasm [51].

*(2) Pur α*. Another protein that has been identified as a HRE-RNA binding is Pur *α* [72]. Involvement of Pur *α* in the pathogenesis of ALS/FTD is supported by experiments showing that Pur *α* overexpression alleviates HRE-mediated toxicity. In other words, Pur *α* overexpression prevents cell death both in mammalian cells and in *Drosophila* [72]. Pur *α* is a component of the RNA-transport granules—particles that carry mRNAs to the nerve fibers where translation of those mRNAs into proteins occurs [74, 75]—and is involved in the regulation of the cell cycle [76] and in cell differentiation [77, 78]. Sequestration of Pur *α* by the *C9orf72* hexanucleotide RNA repeats could impair neuronal mRNA transport thus leading to neurodegeneration [79].

*(3) hnRNPs*. The largest group of proteins sequestered by the *C9orf72* HRE-containing RNA are heterogeneous nuclear ribonucleoproteins (hnRNPs) [44, 51]. It was shown that *C9orf72* interacts with hnRNP-U, hnRNP-F, hnRNP-K [51], hnRNP-A2/B1, and hnRNP-A1 [28], while hnRNP A1 and hnRNP H colocalize with RNA foci [70, 71]. HnRNP H is involved in the regulation of RNA processing [80]; thus, hnRNP H sequestration in RNA cause aberrant RNA processing and may enhance neurodegeneration [70].

*(4) ADARB2*. An RNA-editing enzyme ADARB2 also interacts with the HRE-containing RNAs and colocalizes with RNA foci in C9ALS cases [69]. Although the mechanism by which ADARB2 sequestration in RNA foci could contribute to HRE-mediated toxicity is unclear, it was proposed that this protein may be important for RNA foci formation because ADARBP knockdown results in the reduction of neurons that contain RNA foci [69].

*(5) SRSF1 and SRSF2*. It was shown that serine-arginine-rich splicing factor 1 (SRSF1) and SRSF2 colocalize with RNA foci [70]. Given that SRSF2 is a marker of nuclear speckles, nuclear regions important for storage of splicing factors [81], its accumulation in RNA foci could disrupt the function of these speckles and lead to aberrant RNA processing [73]. Additionally, binding of the nuclear export adaptor SRSF1 to *C9orf72* repeats promotes export of *C9orf72* repeats from the nucleus. As it was demonstrated that *SRSF1* knockdown in C9FTD/ALS *Drosophila* model blocks neurodegeneration, *SRSF1* could be a potential therapeutic target [82].

*(6) ALYREF*. Because aberrant nuclear transport is observed in different C9FTD/ALS models [31–33], the observation that ALYREF (Aly/REF export factor) also colocalizes with RNA foci in C9FTD/ALS cases [70, 73, 83] is of interest. However, although knockdown of nuclear export adaptor *SRSF1* blocks neurodegeneration in *Drosophila*, only a modest decrease in neurodegeneration was observed after knockdown of *ALYREF* [82].

In conclusion, toxic gain-of-function due to aberrant HRE-containing RNA can lead to dysfunctional RNA processing [73], aberrant translation [84], nucleolar stress [51], disrupted nucleocytoplasmic transport [31], and dysfunction in granule transport [85]. Additionally, it was observed that higher abundance of RNA foci in the frontal cortex of C9FTD patients is associated with earlier disease onset [68]. However, several experimental studies failed to detect neurodegeneration in *in vivo* C9FTD/ALS models displaying RNA foci (Table 2). Neurodegeneration was not observed in *Drosophila* models carrying 160 G4C2 repeats [86], 288 G4C2 repeats [87], and 1000 G4C2 repeats [88]. More precisely, *Drosophila* models carrying “RNA-only” repeats that formed RNA foci but not DPRs failed to produce neurodegeneration, while *Drosophila* models carrying “pure repeats” that could form both RNA foci and DPRs displayed neurodegeneration [87, 88]. However, two transgenic C9FTD/ALS mouse models with both RNA and protein toxic gain-of-function failed to produce neurodegeneration [89, 90], while two other transgenic mouse models showed signs of neurodegeneration [91, 92]. Regarding the latter two C9FTD/ALS mice models, whether neurodegeneration was caused by toxic HRE-containing RNAs or DPRs translated from hexanucleotide repeat RNA remains to be assessed [91, 92].

### 6.3. Toxic Gain-of-Function through Accumulation of Dipeptide Repeat Proteins Translated from *C9orf72* HRE-Containing RNA

RNA transcripts of *C9orf72* HRE region can undergo repeat-associated non-ATG (RAN) translation in which different DPR proteins are synthesized [43, 44, 93]. Poly-Gly-Ala, poly-Gly-Pro, and poly-Gly-Arg are translated from different open reading fragments on the sense transcript and poly-Gly-Pro, poly-Pro-Arg, and poly-Pro-Ala from the antisense transcript. Toxicity of DPR proteins seems to be mainly dependent on arginine-containing DPR proteins, particularly poly-Pro-Arg [33, 87, 94, 95]. Arginine-rich DPRs appear to disrupt primarily nucleocytoplasmic transport [33] and RNA processing [33, 95], which can cause dysregulation of translation [96, 97], nucleolar stress [98], disturb ubiquitin proteasome system [99], affect the formation of stress granules [100], and influence the Notch signalling pathway [101]. Additionally, it was shown that poly-Gly-Ala DPR proteins can disturb the ubiquitin proteasome system and cause endoplasmic reticulum stress [102], and enhance the formation of toxic amyloid fibrils [103].

Although there is molecular evidence of DPR proteins' toxicity in *in vitro* and *in vivo* C9FTD/ALS models [104, 105], post mortem analyses of human brain revealed inconsistent results (Table 2). No correlation of DPR proteins with clinical phenotype, severity of disease, and neurodegeneration was observed, and the abundance of DPR proteins was low in the brain regions most affected in ALS and FTD [106–109]. This lack of correlation may signal that neurons carrying toxic DPR proteins are possibly dead at the time of autopsy [18]. Additionally, there were discrepancies in the outcome of the studies comparing the distribution of DPR proteins between ALS and FTD cases [107, 110, 111].

## 7. Cellular Processes Affected in C9FTD/ALS

Although many cellular processes are affected by *C9orf72* repeat expansion, including translation [96], ubiquitin proteasome system [99], Notch signalling pathway [101], granule transport [85], and normal function of TDP-43 (for review see [17]), here we discuss the cellular processes affected by *C9orf72* repeat expansion for which sufficient information is available.

### 7.1. Effects of DPR Proteins and HRE-Containing RNAs on Nucleocytoplasmic Transport

A hallmark morphological feature of TDP-43 proteinopathies is the cytoplasmic accumulation and aggregate formation of TDP-43. We have demonstrated earlier that impaired nucleocytoplasmic transport plays a major role in this process [112]. Importantly, *C9orf72* repeat expansion compromises nucleocytoplasmic transport of proteins and RNA through the nuclear pores. A study in yeast expressing arginine-rich DPR constructs demonstrated that one of the main targets of toxic DPR proteins is nucleocytoplasmic transport [33]. In support of these findings, genetic screens in *Drosophila* designed to identify genes linked to HRE-induced toxicity also identified components of the nuclear pore complex and nucleocytoplasmic transport machinery [31, 32]. Furthermore, RCC1, which is a human protein required for nucleocytoplasmic transport, is mislocalized in cells derived from patients with the *C9orf72* HRE, providing preliminary evidence that C9FTD/ALS HRE might affect nucleocytoplasmic transport in human cells as well [33]. Taken together, the studies in yeast and *Drosophila* provided the first evidence that *C9orf72* repeat expansion causes degeneration by impairing nucleocytoplasmic transport of proteins and RNA [31–33]; however, the effect of C9FTD/ALS HRE on nucleocytoplasmic transport in human cells needs to be further investigated. These findings open a possibility of targeting nucleocytoplasmic transport as a potential new therapeutic target in ALS and FTD [31, 32]. Additionally, as mRNA export factor ALYREF [70, 73, 83] and Ran GTPase-activating protein (RanGAP, regulator of nucleocytoplasmic transport) [31] colocalize with RNA foci (observed in C9FTD/ALS brains) and their function is disrupted, it seems that not only DPRs can lead to aberration of nucleocytoplasmic transport but also HRE-containing RNAs.

### 7.2. *C9orf72* Hexanucleotide Repeat Expansion and RNA Processing

Several studies reported different transcriptional profiles between C9FTD/ALS patients and controls [54, 69, 71, 113–115]. Additionally, Cooper-Knock et al. observed an enrichment in RNA splicing factors in C9FTD/ALS patients [116], further supporting the observation of aberrant RNA processing caused by pathological processes in C9FTD/ALS. The main mechanism that leads to disturbed RNA processing in C9FTD/ALS patients is through sequestration of RBP in RNA foci [14, 73], but it could be also caused by DPR proteins as they can bind to RPB too [33, 95].

### 7.3. HRE-Containing RNAs and Dipeptide Repeat Proteins Can Lead to Nucleolar Stress

The way by which nucleolin binding to RNA foci disrupts the normal function of the nucleolus [51] was discussed above. It was also observed that DPR proteins can cause nucleolar stress in cell lines [98]. Enlargement of the nucleolus was observed in *in vitro* [94, 98] and *in vivo* [101] C9FTD/ALS models expressing arginine-rich DPR proteins. Enlargement of the nucleolus in these models leads to its fragmentation and decreased maturation of rRNA [98].

### 7.4. The Effect of DPRs on Formation and Function of Membraneless Organelles

Cells possess several RNA and protein-containing membraneless organelles, collectively referred to as ribonucleoprotein (RNP) granules, which separate from the cytoplasm or nucleoplasm into a distinct liquid phase-like state that is typically slightly denser than the surrounding [117]. Examples of such organelles are nucleoli and Cajal bodies in the nucleus and processing bodies (P-bodies), stress granules, and transport granules in the cytoplasm [117–119]. Formation of such compartments is triggered by intrinsically disordered low complexity polypeptide sequences present within RBP, which have the ability to undergo phase transitions [120–122]. Furthermore, it has been shown that nuclear membraneless suborganelles, such as Cajal bodies and nuclear speckles, can be nucleated by several coding and noncoding RNAs through the recruitment of proteins residing in these nuclear bodies [123]. Stress induces the formation of many membraneless compartments [124]. Lee et al. reported that arginine-rich DPRs bind low complexity polypeptide sequences of RBP that are components of membraneless organelles. Arginine-rich DPRs alter phase separation of those proteins and, in that way, disturb the function of membraneless organelles [125]. Liquid-liquid phase separation of RBP is very important for the normal formation of membraneless organelles. Other authors also showed that arginine-rich DPRs disturb the function and dynamics of membraneless organelles [126], more precisely, stress granules [98, 100]. Because it has been proposed that stress granules could be seeding points where aggregation of pathological proteins in FTD and ALS begins [127, 128], understanding the influence of DPR proteins on dynamics of membraneless organelles is highly relevant in this context.

### 7.5. *C9orf72* HRE Affects Autophagy and Apoptosis

Adulterations in the autophagic pathway can alter protein homeostasis, causing detrimental effects on neurons that have been implicated in neurodegenerative diseases like Parkinson's disease and tauopathies. Autophagy prevents proteotoxic cell death by removing damaged, misfolded, and unwanted proteins [129]. Hindrance of C9orf72 function leads to a consequential decrease in Rab GTPase activity, a protein involved in membrane fusion, vesicle formation, and vesicle trafficking, which impairs the endocytosis process and autophagy. This causes an increased amount of p62/SQSTM1 and TDP-43, known markers of ALS-FTD [36]. Using C9orf72-deficient mice and cell lines, Sullivan et al. demonstrated that C9orf72 forms a binding complex with SMCR8 and WDR41, which allow it to interact with FIP200/Ulk1/ATG13/ATG101 complex and initiate autophagy [35]. Additionally, C9orf72 HRE have been implicated in increased endoplasmic reticulum stress by causing dysregulation of its calcium channels that leads to neuron apoptosis. This has been quantified by observing a decrease in antiapoptotic genes Bcl-2 and BcL-X_L_, while causing an increased expression of proapoptotic gene BAK [130].

### 7.6. *C9orf72* HRE and Neuroinflammation

Experimental studies investigating C9orf72 loss-of-function in *C9orf72*-deficient mice revealed an increase in proinflammatory cytokines, lymphadenopathy, splenomegaly, alterations in myeloid cells from spleen, and in some cases of autoimmunity [40, 56, 57]. Additionally, a human autopsy study showed that C9ALS cases had more severe microglial pathology in the medulla and motor cortex than ALS cases without *C9orf72* HRE [131]. These findings raised questions about increased neuroinflammation in C9FTD/ALS patients and possible involvement of activated microglia in disease pathogenesis. It was proposed that microglia could represent a link between three potential pathological mechanisms of *C9orf72* HRE, whereby microglia is being most affected by loss of C9orf72 function, while neurons are most affected by a gain-of-function mechanism. Altogether, increased neuroinflammation, accumulation of RNA foci, and DPRs could lead to neuronal death (reviewed in [132]).

## 8. Potential Therapeutic Approaches

Potential therapeutics most commonly target HRE-containing RNA, because its degradation abolishes RNA toxicity and the formation of DPR proteins by RAN translation. The following approaches have been considered so far.

### 8.1. Antisense Oligonucleotides

Antisense oligonucleotides (ASOs) targeting HRE-containing RNAs were tested in different studies [54, 69, 91]. After treatment of C9FTD/ALS cells in *in vitro* [54, 69, 71] and *in vivo* models with ASOs (mice expressing bacterial artificial chromosome (BAC) with the human expanded *C9orf72* gene [91] and adeno-associated virus (AAV) (G_4_C_2_)_66_ mice [133]), a reduction in RNA foci was observed. Additionally, treatment with ASOs also led to a decrease in DPRs [91, 133]. Although ASOs used in these studies did not affect the levels of normal C9orf72 protein [54, 71, 91], the possibility of total silencing C9orf72 cannot be confirmed, so these approaches need to be considered with caution. A clinical trial for ASO targeting HRE-containing RNAs (WVE-3972-01; https://adisinsight.springer.com/trials/700291284) is expected in the fourth quarter of 2018.

### 8.2. Small Molecules

The advantage of using small molecules as therapeutics for C9FTD/ALS is that these compounds could not only target HRE-containing RNAs or DPR proteins but also cellular processes affected by pathology underlying C9FTD/ALS. Su et al. developed three small molecules that target hexanucleotide repeat region of RNAs and could stop RAN translation in (G4C2)_66_-expressing COS7 cells [134]. As epigenetic alterations were observed in C9FTD/ALS cases, epigenetic changes in the *C9orf72* gene were also targeted. Usage of G-quadruplex-binding small molecules yielded increased expression of C9orf72 protein [135–137].

## 9. The Mechanisms of *C9orf72* HRE-Mediated Neurodegeneration Are Not Mutually Exclusive

It is possible that the proposed mechanisms of C9FTD/ALS coexist and act in a combined manner. Maharjan et al. suggested that HRE in *C9orf72* gene affects normal expression of *C9orf72*, diminishes levels of C9orf72 protein, and consequently impairs the formation of stress granules during the cellular stress (caused by formation of RNA foci and DPRs). In other words, *C9orf72* loss-of-function made cells more sensitive to toxicity caused by gain-of-function mechanisms [39]. Additionally, Lall and Baloh [132] proposed a model that unifies all three pathological mechanisms mentioned above. Other authors stressed the importance of generation of rodent experimental models in which all three mechanisms coexist, for example, by crossing *C9orf72* knockout mouse and mouse carrying BAC with human expanded *C9orf72* gene [18]. Although it was shown that ASO targeting HRE-containing RNAs do not affect the levels of normal C9orf72 protein [54, 71, 91], the possibility of silencing *C9orf72* totally should not be overlooked. As ASO targeting HRE-containing RNAs moves into phase 1 of clinical trials, the development of novel cellular and animal experimental models that exhibit all three pathological mechanisms is highly relevant.

## 10. Conclusions

Seven years after the discovery of hexanucleotide repeat expansion in *C9orf72* gene, a lot of progress has been made in the clarification of molecular mechanisms through which *C9orf72* repeat expansions cause neurodegeneration. Three possible, not mutually exclusive, mechanisms could together contribute to the pathogenesis of disease [39]. The majority of the studies investigating the molecular mechanisms of pathological processes in C9FTD/ALS support either toxic HRE-RNA or DPR-dependent gain-of-function, with many studies supporting both mechanisms (Table 1). Hence, to better identify potential therapeutic targets, further studies are needed to fully understand molecular events underlying pathological processes in C9FTD/ALS.

## Figures and Tables

**Table 1:** Summary of studies on the mechanisms by which *C9orf72* repeat expansions cause neurodegeneration.

Reference	Molecular mechanism supported by the study			Experimental model
	Loss-of-function	Gain-of-function		
		RNA-mediated gain-of-function	Protein-mediated gain-of-function	
Renton et al. [15]		+		Human DNA Human brain tissue Cell culture

DeJesus-Hernandez et al. [14]	+	+		Human DNA Human brain tissue Cell culture

Gijselinck et al. [16]	+			Human brain tissue Human DNA

Fratta et al. [58]		+	+	Human DNA

Therrien et al. [52]	+			*Caenorhabditis elegans*

Belzil et al. [135]	+			Human brain tissue Cell culture

Gendron et al. [67]		+	+	Human DNA Human brain tissue Cell lines

Zu et al. [93]		+	+	Human brain tissue Human RNA Cell culture

Mizielinska et al. [68]		+		Human brain tissue

Lee et al. [70]		+		Human brain tissue Cell culture Zebrafish embryos Rat brain

Sareen et al. [71]		+		iPSC-derived neurons

Xu et al. [72]		+		Cell culture *Drosophila*

Lagier-Tourenne et al. [54]		+		Human brain tissue Cell culture

Donnelly et al. [69]		+		Human brain tissue Cell culture iPSC-derived neurons

Ciura et al. [53]	+			Zebrafish Human brain tissue Cell culture

Wen et al. [94]		+	+	Cell culture *Drosophila*

Liu et al. [50]		+	+	Human autopsy tissue Human DNA Cell culture

May et al. [138]			+	Human brain samples Cell culture

Zhang et al. [102]			+	Human brain samples Cell culture

Cooper-Knock et al. [73]		+		Human brain samples Cell culture

Su et al. [134]		+	+	Cell culture

Kwon et al. [95]			+	Cell culture

Haeusler et al [51]		+		Human brain tissue iPSC-derived neurons Cell culture

Mizielinska et al. [87]		+	+	*Drosophila*

Russ et al. [139]		+	+	Human DNA

Prudencio et al. [113]		+	+	Human brain tissue

Chew et al. [140]		+	+	Mouse model expressing 66 G4C2 repeats

Freibaum et al. [32]	+		+	*Drosophila*

Yang et al. [101]			+	*Drosophila* Human brain samples Cell culture iPSC-derived neurons

Jovičić et al. [33]			+	Yeast cells Cell culture

Yamakawa et al. [99]			+	Cell culture Mice brains

Hu et al. [141]		+		Cell culture

Gendron et al. [110]			+	Human brain samples

Tao et al. [98]			+	Cell culture

Tran et al. [86]			+	*Drosophila* iPSC-derived neurons Human brain tissue

Koppers et al. [55]		+	+	Conditional C9orf72 knockout mouse model

Cooper-Knock et al. [83]		+		Human brain tissue

Zhang et al. [31]		+	+	*Drosophila* iPSC-derived neurons Human brain tissue

Rossi et al. [84]		+		Cell culture

Cooper-Knock et al. [116]		+		Cell culture

Schweizer Burguete et al. [85]		+		Cell culture *Drosophila* iPSC-derived neurons

Liu et al. [92]		+	+	BAC mouse model of C9FTD/ALS

Kanekura et al. [96]			+	Human brain samples Cell culture

Boeynaems et al. [142]			+	*Drosophila*

Chang et al. [103]			+	Cell culture

Flores et al. [143]			+	Cell culture

Lin et al. [126]			+	Cell culture

Lopez-Gonzalez et al. [144]			+	iPSC-derived neurons

Liu et al. [145]	+			Human DNA and RNA

Dodd et al. [146]		+		

Zhang et al. [147]			+	Mice that exhibit poly(GA) pathology

Westergard et al. [148]			+	iPSC-derived neurons Cell culture

Sellier et al. [36]	+			Cell culture Zebrafish

Mori et al. [149]		+	+	Human brain samples Cell culture

Gijselinck et al. [19]	+			Human DNA

Ugolino et al. [150]	+			C9orf72 knockout mice C9orf72 knockdown cell models

Lee et al. [125]			+	*Drosophila* Cell culture

O'Rourke et al. [40]		+	+	C9orf72 +/− and C9orf72 −/− mice

Atanasio et al. [56]		+	+	C9orf72-deficient mouse

Jiang et al. [91]		+	+	C9orf72 +/− and C9orf72 −/− mice Mice expressing BAC with the human expanded *C9orf72* gene

Webster et al. [34]	+			Cell culture iNeurons (obtained by differentiation of iNPCs)

Ji et al. [151]	+			C9orf72 knockout mouse model Cell culture

Ohki et al. [152]			+	Zebrafish

Liu et al. [153]		+		Cell culture

Hu et al. [154]		+		Cell culture

Green et al. [155]			+	Cell culture

Schludi et al. [156]			+	Transgenic mice expressing codon-modified (GA)_149_

Gupta et al. [157]			+	Cell culture

Khosravi et al. [158]			+	Cell culture

Shi et al. [159]			+	Cell culture

Saberi et al. [160]			+	Human brain samples

Kramer et al. [105]			+	Cell culture

Herranz-Martin et al. [161]		+	+	Mice that overexpress 10 or 102 interrupted G4C2 repeats

Zhou et al. [162]			+	Cell culture Human brain samples

Lehmer et al. [163]			+	Human CSF

Hautbergue et al. [82]		+	+	*Drosophila* Cell culture

Boeynaems et al. [100]			+	Cell culture

Maharjan et al. [39]	+	+	+	Cell culture

Moens et al. [88]		+	+	*Drosophila*

Cheng et al. [164]			+	Cell culture

Swinnen et al. [165]		+		Zebrafish

Shi et al. [59]	+		+	Cell culture iPSC-derived neurons Human brain samples

Simone et al. [137]		+	+	iPSC-derived neurons *Drosophila*

Tabet et al. [166]			+	Human brain sections Cell culture

Corrionero and Horvitz [167]	+			*Caenorhabditis elegans*

Zamiri et al. [168]		+		CD spectroscopy UV melting Gel electrophoresis

Swaminathan et al. [104]			+	Zebrafish

Yeh et al. [169]	+			Zebrafish

Meeter et al. [170]			+	Human brain samples
Nonaka et al. [171]			+	Cell culture

Frick et al. [29]	+			Human brain samples Cell culture *C9orf72* knockout mice

BAC: bacterial artificial chromosome; CSF: cerebrospinal fluid; iNPCs: induced neural progenitor cells; iPSC: induced pluripotent stem cells. Studies investigating pathological mechanisms of *C9orf72* HRE but not clearly supporting any of the three proposed disease mechanisms are not included in this table. Search for these studies was completed on May 20, 2018. The literature search was performed in Google Scholar using the keywords: “C9orf72”, “mechanism”, “pathological”, “loss-of-function”, “gain-of-function”, “haploinsufficiency”, “RNA foci”, and “DPR”. The literature search was conducted by two independent researchers.

**Table 2:** Evaluation of potential mechanisms underlying pathology in C9FTD/ALS.

Molecular mechanism underlying pathology in C9FTD/ALS	Pros	Cons
Loss-of-function	*C9orf72* loss-of-function models in *C. elegans* and zebrafish result in motor neuron degeneration	*C9orf72* loss-of-function mouse models do not show phenotype characteristic for ALS and FTD
	Carriers of *C9orf72* HRE have decreased levels of C9orf72 mRNA and proteins in the brain	Patients homozygous for *C9orf72* repeat expansion do not have more severe symptoms of disease

RNA-mediated gain-of-function	HRE-containing RNA transcripts accumulate and form nuclear aggregates, or RNA foci, in the brain of patients with mutated *C9orf72*	*Drosophila* models of RNA toxic gain-of-function fail to produce neurodegeneration
	Sequestration of RNA-binding proteins into RNA foci can disrupt RNA processing, translation, nucleocytoplasmic transport, and granule transport and lead to nucleolar stress	The results on RNA toxic gain-of-function mouse models are conflicting and need to be further investigated
	Higher abundance of RNA foci in patients carrying C9FTD/ALS HRE is associated with earlier disease onset	

Protein-mediated gain-of-function	*Drosophila* model of protein-mediated gain-of-function develops neurodegeneration	Amounts of DPR in the brain do not correlate with clinical phenotype, severity of diseases, and neurodegeneration
	DPR disrupt nucleocytoplasmic transport, RNA processing, translation, ubiquitin proteasome system, formation of stress granule, and Notch signalling pathway and can lead to nucleolar stress	Abundance of DPR is low in the brain regions most affected by ALS and FTD

ALS: amyotrophic lateral sclerosis; C9FTD/ALS: hexanucleotide repeat expansion in C9orf72 causing ALS and FTD; DPR proteins: dipeptide repeat proteins; FTD: frontotemporal dementia; HRE: hexanucleotide repeat expansion.

## Abbreviations

ALS: Amyotrophic lateral sclerosis
ALS-bi: ALS with behavioral impairment
ALS-ci: ALS with cognitive impairment
ALS-cbi: ALS with combined cognitive and behavioral impairment
ALYREF: Aly/REF export factor
ASOs: Antisense oligonucleotides
BAC: Bacterial artificial chromosome
bvFTD: Behavioral variant of frontotemporal dementia
C9orf72: Chromosome 9 open reading frame 72
C9FTD/ALS: Hexanucleotide repeat expansion in *C9orf72* causing ALS and FTD
CSF: Cerebrospinal fluid
DENN proteins: Differentially expressed in normal and neoplasia proteins
DPR proteins: Dipeptide repeat proteins
FTD: Frontotemporal dementia
FTLD: Frontotemporal lobar degeneration
FUS: Fused in sarcoma
G4C2: GGGGCC
GEF: Guanine exchange factor
hnRNPs: Heterogeneous nuclear ribonucleoproteins
HRE: Hexanucleotide repeat expansion
IBMFD: Inclusion body myopathy with frontotemporal dementia
iNPCs: Induced neural progenitor cells
iPSC: Induced pluripotent stem cells
MND: Motor neuron disease
NCL: Nucleolin
NPC: Nuclear pore complex
P-bodies: Processing bodies
PNFA: Progressive nonfluent aphasia
pTDP-43: Phosphorylated TDP-43
RAN translation: Repeat-associated non-ATG translation
RanGAP: Ran GTPase-activating protein
RBP: RNA binding proteins
RNP: Ribonucleoprotein
SD: Semantic dementia
SRSF: Serine-arginine-rich splicing factor
TDP-43: TAR DNA-binding protein 43
VCP: Valosin-containing protein.
